# Colorectal adenocarcinoma in children and adolescents: the management of advanced disease

**DOI:** 10.1007/s00384-025-05017-2

**Published:** 2025-11-19

**Authors:** Riccardo Guanà, Ana Sofia Soto Torselli, Francesco De Leo, Benedetta Marino, Silvia Perin, Giada Morgani, Marco Ettore Allaix, Matilde Piglione, Valentina Di Martino, Enrico Costantino Falco, Fabrizio Gennari

**Affiliations:** 1 Pediatric Surgery, Turin University, Turin, Italy; 2 Pediatric Surgery Unit, Regina Margherita Children’s Hospital, Turin, Italy; 3 Pediatric Surgery Unit, Department of Women and Children’s Health, Padua General Hospital, Padua, Italy; 4 Department of Surgical Sciences, University of Torino, Turin, Italy; 5 Paediatric Hematology-Oncology Unit, Department of Paediatrics, Regina Margherita Children’s Hospital, Turin, Italy; 6 Pathology Unit, University Hospital of Health and Science, Turin, Italy; 7 Division of Pediatric General, Thoracic & Minimally Invasive Surgery, Regina Margherita Children’s Hospital, Piazza Polonia 94, 10126 Turin, Italy

**Keywords:** Colorectal adenocarcinoma, Children, Adolescents, Surgery

## Abstract

**Introduction:**

Colorectal carcinoma (CC) is a rare disease in the pediatric population, with an annual incidence of 1 in 10 million adolescents, and it accounts for approximately 1% of pediatric solid neoplasms. It is the most common primary gastrointestinal malignancy in children with the vast majority of CCs being adenocarcinoma (CA). Unfortunately, the proportion of poorly differentiated, mucinous type, signet-ring cell containing carcinomas is higher in younger patients than in adults. Moreover, due to the low awareness of the disease, diagnosis is usually delayed until advanced stages, resulting in an extremely poor prognosis. Surgery is the only curative modality for localized CAs, whereas adjuvant chemotherapy is the standard of care for patients with stage III cancer to eradicate micro-metastases.

**Patients and methods:**

In the last 10 years, we treated 3 patients diagnosed with CA: a 14-year-old female, a 15-year-old male, and a 15-year-old female. All patients presented to our Emergency Department with nonspecific symptoms of abdominal pain and vomiting.

**Results:**

All patients were subjected to laparoscopic tumor resection to relief intestinal obstruction. In the male patient, laparoscopy was converted to laparotomy to safely assess the anatomy because of strong peritoneal adhesions. No stomas were created, in order to improve quality of life. Oxaliplatin and 5-fluorouracil-based regimens were among the most commonly used chemotherapy combinations. The 15-year-old female and the 15-year-old male died 1 year after the surgical resection, while the 14-year-old female is still on follow-up.

**Conclusions:**

CAs behave aggressively in children; they not only show a poorer response to chemotherapy, but are also associated with extensive intramural spread and peritoneal carcinomatosis. Lack of awareness and timely intervention remain the main challenges for early diagnosis and improved prognosis of CA.

## Introduction

Colorectal carcinoma (CC) is very rare in the pediatric population, with an annual incidence of less than 1 case in 10 million adolescents [1]. Although colonic adenocarcinoma (CA) is the most common primary gastrointestinal malignancy in children, it accounts for less than 1% of the pediatric neoplasms.

Due to the high presence of poorly differentiated tumors with a highly aggressive nature and a low index of suspicion in this population, diagnosis is usually delayed until the disease reaches an advanced stage, resulting in a very poor prognosis. Symptoms in children may be nonspecific, ranging from mild abdominal discomfort to acute bowel obstruction, the latter occurring more frequently in pediatric patients than in adults [2]. Ninety percent of pediatric CC’s occur sporadically, with only a small number of cases associated with predisposing conditions [3].

Molecular genetic techniques, including microarray analysis, have been described in the literature as providing promising diagnostic, prognostic, and predictive markers of CC in the pediatric population that may reveal future targets for therapy.

At present, radical surgical resection of the affected bowel, lymphadenectomy, and excision of the infiltrated organs remain the mainstay of therapy. In advanced stages, adjuvant chemotherapy may be required to eradicate micro-metastases, possibly in combination with local radiotherapy or targeted therapies.

Here, we present the cases of three patients treated for CA at our pediatric center over the past 10 years (Table 1): two females and one male, with a median age at diagnosis of 15 years. In all the cases, the patients presented at our pediatric Emergency Department (ED) with vague abdominal symptoms, and further diagnostics prompted surgical exploration and tumor excision. Adjuvant chemotherapy was necessary in all presented cases.

**Table 1:** Summary of the patients’ data

Patient	Age (years)	Gender	Family history	Symptoms	Diagnosis	Location	Surgery	Staging	Chemotherapy (first line)	Chemotherapy (second line)	FU
#1	14	F	/	Abdominal pain	Endoscopic	Hepatic flexure	Right hemicolectomy (laparoscopy)	pT4N2Mx	FOLFOX (6 months)	/	Disease-free
#2	15	F	Intestinal neoplasia in grandmother	Abdominal pain	Endoscopic	Right transverse colon	Right hemicolectomy (open)	pT4bN2b	FOLFOX (8 cycles)	FOLFOX + bevacizumab	Died
#3	15	M	**/**	Abdominal pain	Endoscopic	Descending colon	Left hemicolectomy (open)	pT4aN2bM1c	FOLFOX + cetuximab	Cetuximab + 5-FU	Died

To date, only one patient is still in follow-up, disease-free, while two patients died about 1 year after the initial diagnosis, due to disease recurrence.

In consideration of the aggressive nature of CAs in children, with a low chemo-sensitivity and a high rate of intramural spread and peritoneal carcinomatosis, prompt diagnosis and early surgical intervention, with hemicolectomy and tumor resection, remain the standards of care to improve the outcome and future prognosis.

This study adhered to the reporting recommendations indicated by CARE (CAse REports guidelines).

## Case report #1

A previously healthy 14-year-old female presented to our ED with a 20-day history of vague abdominal pain and vomiting. No predisposing medical or family history was recorded. Upon evaluation, an abdominal ultrasound examination showed the presence of a solid mass in the right hypochondrial area. Further evaluation with a colonoscopy and an abdominal CT scan showed marked distension of the intestinal loops up to the hepatic flexure of the colon, where a stenotic area caused by an endoluminal solid mass was observed. The pathology report of endoscopic biopsies was positive for colonic adenocarcinoma with signet-ring cells, prompting a laparoscopic right hemicolectomy. Clear surgical resection margins were obtained, with no macroscopic metastases or peritoneal infiltration. After histopathological and immunohistochemical analysis (positive to antiCDX2, ck7, and AE1-AE3), the tumor was classified as stage T4N2Mx according to the TNM anatomical pathology classification system. After surgery, the patient received adjuvant chemotherapy according to the FOLFOX protocol for a period of 6 months. At the end of the chemotherapy, no residual lesions were detected, and after 10 years of follow-up, the patient is disease-free.

## Case report #2

A 15-year-old female was referred to our pediatric ED with a month-long history of asthenia, abdominal pain, and nausea. Family history was notable for intestinal neoplasia in a paternal grandmother and aunt. Blood tests showed moderate anemia, and stool examination was positive for occult blood. Upon evaluation, abdominal ultrasound and X-rays appeared normal. A subsequent CT scan and colonoscopy revealed circumferential wall thickening of the right transverse colon extending for approximately 5 cm, causing an important stenosis of the lumen. Histological evaluation of endoscopic biopsies was compatible with infiltrating signet-ring adenocarcinoma. The patient underwent surgical exploration with right hemicolectomy for the resection of the colonic mass, which involved the serosa, the peritoneum, and the greater omentum. The tumor was graded stage 4 pT4bN2b, according to the TNM anatomical pathology classification system. Molecular analysis of the specimens showed preserved nuclear expression of NRAS, KRAS, BRAF, MSH2, MLH1, and PMS2. The patient subsequently received chemotherapy with the FOLFOX regimen, completing a total of eight cycles. A year and a half after surgery, due to a sudden elevation in the oncological markers level (CEA, CA 19-9), a PET CT scan revealed peritoneal recurrence. Laparoscopic biopsies of the peritoneal nodules confirmed the recurrence. Further chemotherapy with FOLFOX in combination with bevacizumab was administered; however, the patient died 29 months after the initial diagnosis due to disease-related complications.

## Case report #3

**Fig. 1:**
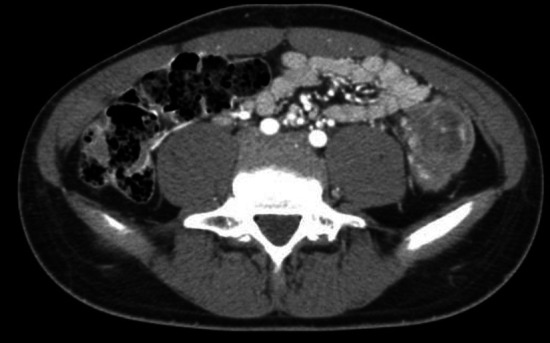
Abdominal CT-scan image demonstrating neoplastic mass in the descending colon which measures 5 × 3 cm

**Fig. 2:**
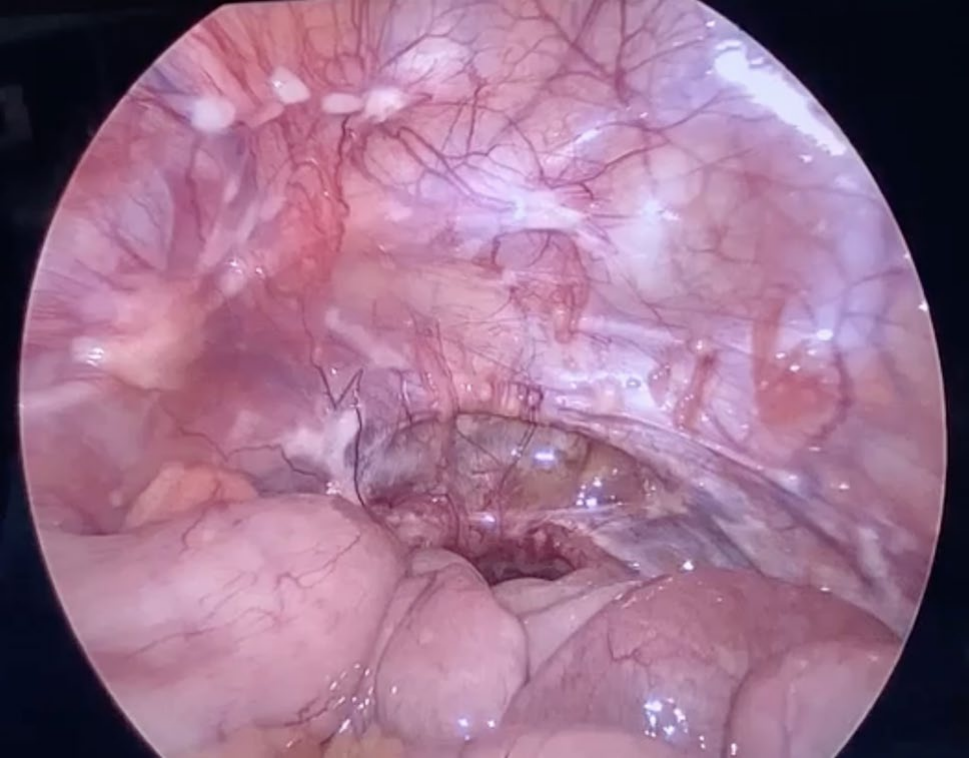
Peritoneal carcinosis observed at laparoscopy

**Fig. 3:**
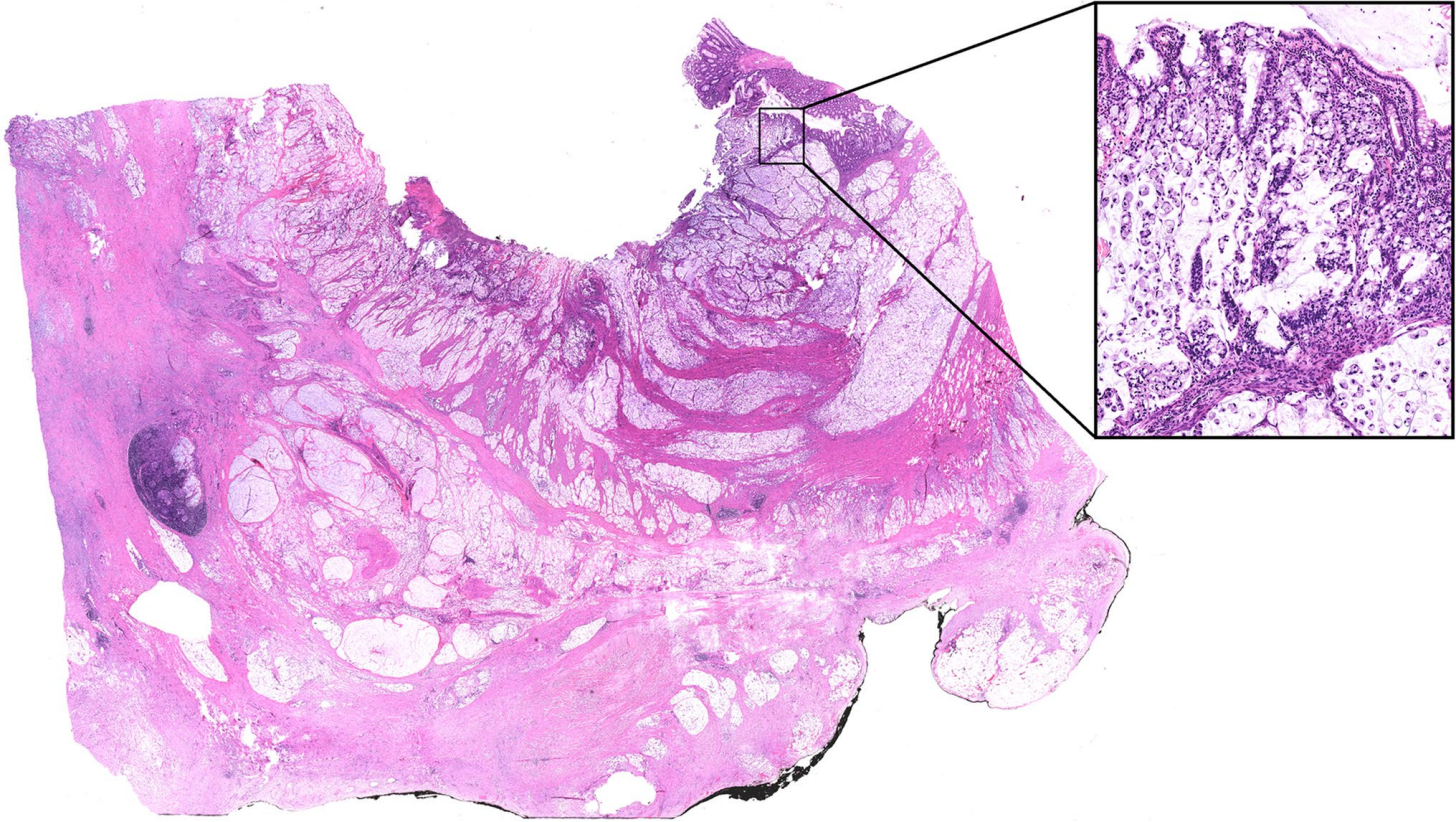
Signet-ring cell carcinoma effacing the colonic wall structure (hematoxylin and eosin, original magnification 40×) with high magnification insert showing signet-ring cells originating from the mucosa and infiltrating the underlying stroma (hematoxylin and eosin, original magnification 300×)

A 15-year-old male presented to our pediatric ED with a 4-month history of abdominal pain. The patient was otherwise healthy, with no prior medical treatments and no reported weight loss. Routine blood tests returned negative, but stool testing revealed occult blood, and fecal calprotectin was elevated. Subsequent colonoscopy and total body CT scan revealed a stenosing lesion in the descending colon measuring 5 × 3 cm (Fig. 1). No mesenteric lymph nodes were observed; however, a millimetric nodule was noted in the left lower lung lobe. Endoscopic biopsies obtained during colonoscopy demonstrated the presence of a poorly differentiated CA with signet-ring cells. PET scan showed no evidence of metastatic disease. Based on preoperative imaging, the patient underwent a left hemicolectomy. Intraoperatively, peritoneal seeding of the tumor and adenopathy was found (Fig. 2). Postoperative pathological staging classified the tumor as a pT4aN2bM1c according to the TNM anatomical pathology classification system. Immunohistochemistry was positive for PD-L1 (CPS 3), while genetic analysis was negative (Fig. 3). The patient was treated with six cycles of FOLFOX plus Cetuzimab. Follow-up abdominal CT scan revealed progression of peritoneal disease, hepatic involvement of the eighth segment, and a small nodule in the left lower lung lobe. Despite an additional six cycles of chemotherapy (cetuximab + 5-fluorouracil), the patient developed ascites requiring percutaneous drainage. Before the initiation of a rescue protocol with capecitabine, oxaliplatin, and bevacizumab, the boy ultimately died 1 year after the initial diagnosis.

## Discussion

Colorectal adenocarcinomas represent more than 95% of CC and originate from the glandular epithelium; they are one of the most common cancers worldwide, with a lifetime risk of about 5% in developed countries. Incidence rises sharply after age 50, but early-onset (< 50 years) and pediatric cases are increasing globally, with a slight prevalence in males [1].

Colorectal carcinoma in the pediatric population is a rare and challenging condition, with an annual incidence of less than one case per 10 million adolescents. Despite being the most common gastrointestinal malignancy in children, CA represents less than 1% of pediatric solid neoplasms [2].

The rarity of the disease in this population, combined with its typically aggressive nature, often leads to delays in diagnosis and poor prognosis.

Hereditary polyposis syndromes (HPS) including familial adenomatous polyposis (FAP), juvenile polyposis syndrome (JPS), and Peutz-Jeghers syndrome (PJS) are all predisposing conditions to colorectal cancer, with a genetic basis (mutations in SMAD4 or BMPR1A genes). The reported risk of developing CC in JPS is about 18%, with high-grade dysplasia being the most common indication for colectomy [7].

In adult CC, multimodal therapy is considered the gold standard and is tailored according to cancer stage.

Surgery is the primary treatment for localized disease, whereas systemic chemotherapy (including capecitabine, fluorouracil, irinotecan, leucovorin, oxaliplatin, trifluridine, and tipiracil) is indicated for advanced stages. Radiation therapy may be employed either internally or externally, while targeted therapy involves monoclonal antibodies against proteins regulating cancer cell proliferation, with bevacizumab and cetuximab being the most commonly used.

Immunotherapy utilizes biological agents such as ipilimumab, nivolumab, and pembrolizumab to enhance tumor-infiltrating lymphocyte activity.

Adjuvant chemotherapy is primarily indicated for stage III (node-positive) and selected high-risk stage II (e.g., poor differentiation, T4 tumors, lymphovascular/perineural invasion, inadequate nodal sampling, obstruction/perforation).

Standard regimens include FOLFOX (5-FU, leucovorin, oxaliplatin), CAPEOX (capecitabine + oxaliplatin), and capecitabine or 5-FU/leucovorin for patients not suitable for oxaliplatin.

The usual duration of therapy is 6 months.

In metastatic disease, systemic chemotherapy remains the backbone of treatment, with commonly used regimens including FOLFOX, FOLFIRI (irinotecan-based), and CAPEOX.

The role of molecular markers in adult CC is increasingly recognized. KRAS/NRAS/BRAF status guides the use of anti-EGFR therapies (e.g., cetuximab) in the metastatic setting, while HER2 amplification and NTRK fusions also represent actionable targets in metastatic CC.

The use of immunotherapy with immune checkpoint inhibitors is biomarker-driven, with the main indications being microsatellite instability–high or mismatch repair–deficient tumors, as well as metastatic disease. Pembrolizumab (PD-1 inhibitor) is the current first-line therapy, while nivolumab alone or in combination with Ipilimumab (PD-1 plus CTLA-4 inhibitors) may be used as an option for patients with progression after chemotherapy [8].

Differently from adults’ experience, where randomized controlled trial studies and guidelines are widely available, the case reports presented in our series reflect the typical clinical challenges associated with pediatric CC.

Our patients presented with nonspecific symptoms, such as vague abdominal pain, nausea, and vomiting, while in adults, CC manifests with persistent changes in bowel habits, such as diarrhea or constipation, hematochezia, abdominal pain, cramps or bloating, and weight loss.

Pediatric CC is usually aggressive and poorly differentiated, meaning that at the time of diagnosis, the disease is often advanced and already metastatic.

This underlines the need for a higher index of suspicion, particularly when teenage patients present with atypical abdominal symptoms and colic solid masses.

The diagnosis of CC relies on both clinical and histological feature; therefore, early tumor grading and staging are critical for improving outcome.

Family history, as documented in one of our cases, raises important considerations regarding the contribution of hereditary factors to pediatric CC.

Although the majority of pediatric CC cases occur sporadically (90%), a genetic predisposition should be considered in patients with a significant family history. This highlights the importance of genetic counseling and screening for inherited conditions in pediatric CC.

The aggressive nature of these tumors, along with their tendency for intramural spread and peritoneal carcinomatosis, requires prompt, comprehensive, and intensive treatment strategies. In particular, worst prognosis’s cases, such as those with peritoneal metastases or extensive multi-organ involvement, necessitate aggressive chemotherapy regimens.

Current treatment remains largely based on radical surgery (hemicolectomy with mass resection, lymphadenectomy, and excision of secondary lesions) [4], while adjuvant chemotherapy, especially, plays a vital role in managing microscopic disease and reducing recurrence. However, while the standard adult chemotherapy regimen (such as FOLFOX) has shown some benefit in pediatric patients, the high rate of chemotherapy resistance in pediatric CC raises significant concern, as highlighted by the poor response in two of our cases.

Recent advances in molecular genetics, including microarray analysis and molecular profiling, have identified potential diagnostic, prognostic, and predictive markers in pediatric CC.

These molecular tools could play a critical role in tailoring therapy and improving outcomes in the future. Furthermore, novel targeted therapies and immunotherapies represent promising therapeutic avenues. Although not yet standard practice in pediatric CC, further studies on the use of PD-L1 inhibitors and other immunotherapies warrant close attention [5, 6].

## Conclusions

Colorectal carcinoma is a rare and aggressive malignancy in the pediatric population that presents significant diagnostic and therapeutic challenges. Early recognition, aggressive surgical treatment, and personalized medical therapies will be crucial in enhancing survival rates and quality of life for these young patients. Ongoing research into molecular markers, genetic profiling, and novel therapies is essential to improve outcomes for this patient population.

## Data Availability

All data supporting the findings of this study are available within the paper. No datasets were generated or analyzed during the current study.
